# Syphilis and Its Correlates among Heterosexual Males Attending Sexually Transmitted Infection Clinics – Observation from a Multicity Cohort in Jiangsu Province, China

**DOI:** 10.1371/journal.pone.0095289

**Published:** 2014-04-17

**Authors:** Xiao-Yan Liu, Chao Hao, Hui Jiang, Lin Sun, Jian-Bo Zhou, Yue-Ping Yin, Weiming Tang, Ning Jiang, Tanmay Mahapatra, Sanchita Mahapatra, Xiang-Sheng Chen, Hai-Tao Yang, Geng-Feng Fu, Xi-Ping Huan

**Affiliations:** 1 Jiangsu Provincial Center for Disease Prevention and Control, Nanjing, China; 2 Changzhou Center for Disease Prevention and Control, Nanjing, China; 3 Yangzhou Center for Disease Prevention and Control, Nanjing, China; 4 National Center for STDs Control, China CDC, Nanjing, China; 5 Department of Epidemiology, School of Public Health, University of California Los Angeles, Los Angeles, California, United States of America; University of Kentucky College of Medicine, United States of America

## Abstract

**Objectives:**

To estimate the prevalence of HIV and syphilis, incidence of syphilis and to identify the correlates of syphilis infection among heterosexual male attendees of sexually transmitted infection (STI) clinics (MSC).

**Methods:**

A cohort study of one-year duration was conducted in Yangzhou and Changzhou cities in Jiangsu province of China. The baseline survey commenced in June 2009, recruited 1225 consenting adult MSCs (609 in Yangzhou and 617 in Changzhou) through STI-clinic based convenience sampling.

**Results:**

Baseline HIV and syphilis prevalence were 0.49% and 17.29% respectively. Syphilis incidence rate was 7.22 per 100 person-years (6.53 in Yangzhou and 7.76 in Changzhou) during the 6-month follow-up with retention fractions of 27.38% and 35.15% for Yangzhou and Changzhou respectively. Majority of the participants were middle-aged, high school educated, married, living with partners and non-migrants. Very few subjects reported recent and consistent condom-use with regular partners. Although considerable number of MSCs reported recent sexual exposure with female sex workers (FSW) and non-FSW casual partners, the proportion of reported condom use was very low during those exposures. In multivariate analyses higher age, having recent sex with FSWs and being HIV-positive were associated with higher syphilis sero-positivity while higher education was protective. In bivariate analyses, being married, divorced/widowed, official residency of the study cities and non-use of condom with regular partners predicted higher risk.

**Conclusions:**

Considering the potential bridging role of MSCs between high and low-risk populations, effective intervention strategies among them targeting the correlates of syphilis infection are urgently called for in Jiangsu province of China.

## Introduction

The emergence of sexually transmitted infections (STI)is currently one of the biggest public health concerns in China [1], [2]. Among these STIs, syphilis is considered to be the infection with most typical epidemic characteristics. Since the invention of penicillin, no country has experienced such a dramatic upsurge of reported syphilis cases, as China is presently having [3], [4]. After having been nearly eliminated from this country more than fifty years ago [4], a rapid re-emergence of syphilis infection has been observed here during the past two decades [2], [5]. Both case report system and cross-sectional studies suggested that China met with an expanding syphilis epidemic in the new century. The alarming nature of the epidemic may be exemplified by the information that syphilis has now become the most commonly reported communicable disease in Shanghai, the largest city of China [3]. Nationwide surveillance data indicated that syphilis prevalence in the general population increased from 0.17 cases per 100,000 in 1993 to 20.0 cases per 100,000 in 2010 [5], [6]. In Jiangsu, this prevalence had already reached 13.0 per 100,000 in 2005 [6].

Despite of having nationwide STI control programs among identified high-risk populations [e.g.: like female sex workers (FSW), men who have sex with men (MSM)] already in place, this upsurge of syphilis epidemic generated considerable public health concern. Identification of other specific population groups having potentials for being high-risk or bridging between high and low-risk populations for the spread of syphilis seemed critically important for planning additional targeted intervention programs to curb this epidemic down. Available evidences in contemporary scientific literatures did show that the burden of syphilis was found to be substantially high among the attendees of STI-clinic worldwide as well as in China. A study conducted at a STI-clinic of Amazon Region of Brazil reported a syphilis prevalence of 9.4% among the participants [7]. Another study conducted in USA reported a sero-positivity of 2.6% among STI-clinic attendees [8]. Prevalence of syphiliswas11.9% among persons visiting STI-clinics in Guangxi province of China [9]. Although these STI-clinic attendees included FSWs and MSM, still in the Guangxi study, syphilis prevalence was pretty high among non-MSM (11.2%) and non-sex-worker (11.3%) population while among male it was 10.2%. Being mostly married and living with partners, majority of the heterosexual STI-clinic attendees who usually engage in sex with their non-sex-worker partners, are thus likely to be a major source for the spread of STI to their regular or casual heterosexual partners [10]. As a result, heterosexual male attendees of the STI-clinics (MSC) are likely to become a bridge population between most-at-risk groups and the general population by playing an important role in the spread of HIV and other STIs [11]. However, only a limited number of studies had ever been conducted involving this high-risk-population [12], [13], [14], few of which examined the factors correlated with syphilis and none of them estimated the incidence rate of STIs in this population in China. Thus dearth of information on the incidence and prevalence of STIs and their socio-behavioral correlates among MSC population in China, called for a longitudinal evaluation to understand their role in the current STI epidemic in this country.

In 2009,“China Mega Project” was implemented in four provinces of China with the aim to evaluate the impact of enhanced STI care on the prevention of HIV and other STIs among high-risk groups (FSW, MSM and MSC). As apart of the project, the objectives of our study were to estimate HIV and syphilis prevalence, the incidence of syphilis as well as to identify the factors correlated with syphilis infection among MSCs, in order to provide guidance to the policy-makers while developing effective intervention strategies specifically targeting this most-at-risk population.

## Methods

### Ethical Statement

The study process and contents were approved by the Ethics Committee of National Center for STD Control of China CDC. Signed informed consent was obtained from each of the participants prior to the interviews and blood collection. Each participant was free to decline his participation or withdraw himself from the study at any point of time.

### Recruitment

This study was a part of the “China Mega Project”. The baseline survey of this study was conducted between June and September 2009 in two cities (Yangzhou and Changzhou) of Jiangsu province of China.

Site [STI-clinics in hospitals, local Center for Disease Control (CDC) etc.] based convenience sampling was used for recruiting the participants in the targeted cities. Biological males, aged 18 years or more, who attended the STI-clinics for seeking health care services during the recruitment period and provided informed consent in favor of voluntary participation in the baseline survey and follow-up were recruited for the study. Persons who were unable to participate actively due to medical reasons or intoxication and those who were engaged in sex with other men in the past three months (as per the definition used in the “China Mega Project”, male attendees of STI clinics, who had female sexual partners and were not engaged in sex with men in last three months, were considered to be heterosexual) were excluded from the survey.

### Biological Sample Collection and Structured Interview

After the assessment of eligibility and collection of informed consents, venous blood sample was collected from each participant for free HIV and syphilis testing. Interviewer administered, face-to-face, structured questionnaire-based interviews were then conducted to collect information on age (continuous or categorized into less than 25, 25 to 39, 40 and above), education level (illiterate or educated up to elementary school, junior or senior high school, college or higher), marital status (single, married and divorced/widowed), residency status (official resident of either of the cities under study, Jiangsu province or other provinces) and some other demographic factors. Besides these, recent sexual behaviors, including the status of condom use in the last vaginal intercourse with FSWs, casual and regular partners, the status of condom use in the previous three months when engaged in vaginal intercourse with FSWs, casual and regular partners were also enquired.

After the interview, following the national guidelines, the testing results were declared to the participants in a private room where the STI-clinic physicians conducted physical examination and provided counseling to each of the participants.

### Follow Up

All the HIV and syphilis negative participants were encouraged to take part in the follow up phase. During this phase, 6 months after the baseline survey, an appointment was scheduled for each participant. In order to minimize the loss to follow up, all the participants were reminded again two weeks prior the scheduled appointment date. During the follow up, same information (those collected at baseline) was collected again from each participant and blood tests were repeated.

The retention fraction was defined as proportional participation in successive rounds relative to the initial round (in each round, positive cases were removed from the denominator). Incidence rates were estimated by using the number of sero-conversions within the follow-up period as the numerator and the total at risk (for HIV or syphilis respectively) person-year within each follow-up period as the denominator. For those who sero-converted, half of the follow-up duration (between two follow-ups) was used as their contribution to the total person time at risk.

### Serologic Measures

Blood samples tested positive for HIV-1 or HIV-2 in the rapid test for screening (using ELISA kit, Livzon Diagnostic Inc., Zhuhai), were subjected to HIV Blot 2.2(MP Biomedicals Asia Pacfic Pte. Ltd, Singapore), for the confirmation of the diagnoses. Samples positive for syphilis screening(using ELISA kit, Wantai Biological Pharmacy Enterprise Co., Ltd, Beijing) were confirmed by Toluidine Red Untreated Serum Test (TRUST, Wantai Biological Pharmacy Enterprise Co., Ltd). Participants diagnosed positive for the Western Blot test for HIV antibody were defined as HIV-positives while persons positive for both Toluidine Red Unheated Serum Test (TRUST: A Qualitative and Quantitative Card Test for the Serologic Detection of Syphilis) and ELISA were defined as currently syphilis positives [15], [16]. Participants diagnosed (through physical examinations and blood testing) to suffer from any STI, were appropriately treated by the STI-clinic physicians. Persons tested positive for HIV were referred to national HIV care and treatment program for further follow up and required treatment.

### Data Analysis

Data entry was done ensuring accuracy by double entry and multiple logic checks using EpiData 3.02. [17]. SAS statistical analysis software version 9.1 [18] was used to describe the demographic and sexual behaviors, to estimate the incidence of syphilis and to measure the prevalence of HIV and syphilis. To identify the correlates of syphilis, we first conducted simple logistic regression procedure to perform univariate analysis [odds ratio (OR) and 95% confidence intervals (95%CI)]. Multiple logistic regression procedure with backward model selection method was then used for the multivariate analysis (variables with a p-value of less than 0.25 were included in the multivariate analysis model).

## Results

This multicity cohort study was conducted over one year since the commencement of the baseline survey from June 2009 till the completion of the follow up duringJune2010. Altogether 1225MSCs from 34 sites (22 in Yangzhou and 12 in Changzhou; 18 STI-clinics of CDC and 16 STI-clinics in hospitals) were recruited for the baseline survey. Meanwhile, 34 persons (15 in Yangzhou and 19 in Changzhou) were excluded either because they had sex with men or they did not want to participate.

Among these participants, 609 (49.71%) were recruited at Yangzhou while the other 617 (50.29%) were surveyed at Changzhou. The observed baseline HIV and syphilis prevalence were 0.49% (95%CI: 0.20, 1.12) and 17.29% (95%CI: 15.25, 19.57) respectively.

About half of the participants were aged between 25 and 40. About 71% of them (about 74% in Yangzhou and 67% in Changzhou)attended junior or senior high school. Majority (77%) were married (85% in Yangzhou and 69% in Changzhou) and approximately 74% of the participants (81.6% and 66.6% for Yangzhou and Changzhou respectively)were currently living with their heterosexual partners. About 84% were the official resident of the cities under study. (Table 1).

**Table 1:** Distribution of socio-demographic factors, sexual behavior, HIV and syphilis sero-positivity among heterosexual male attendees of STI clinics (MSC) in Yangzhou and Changzhou cities of Jiangsu Province, China, at baseline during 2009.

Variable		Yangzhou (n = 609)			Changzhou (n = 617)			Overall		
		n	%	95% CI	n	%	95% CI	n	%	95% CI
Age	Less than 25	66	10.93	8.36–13.31	114	18.48	15.40–21.55	180	14.77	12.85–16.91
	25–40	280	46.35	42.01–49.95	305	49.43	45.48–53.39	585	47.99	45.16–50.84
	40 and above	258	42.72	38.43–46.30	196	31.76	28.08–35.45	454	37.24	34.53–40.04
Education	Illiterate/elementary school	91	14.88	12.10–17.78	81	13.13	10.46–15.80	172	14.05	12.18–16.15
	Junior/senior high school	453	74.38	70.91–77.86	416	67.42	63.71–71.13	869	71.00	68.35–73.51
	College or above	63	10.34	7.92–12.77	120	19.45	16.32–22.58	183	14.95	13.02–17.10
Marital status	Single	76	12.47	9.85–15.11	168	27.23	23.71–30.75	244	19.93	17.75–22.31
	Married	518	85.06	82.22–87.90	426	69.04	65.39–72.70	944	77.12	74.65–79.43
	Divorced or widowed	13	2.13	0.98–3.28	23	3.73	2.23–5.23	36	2.94	2.10–4.09
Living with	None	54	8.87	6.60–11.13	119	19.29	16.16–22.41	173	14.12	12.25–16.23
	Heterosexual partner	497	81.61	78.52–84.69	411	66.61	62.88–70.34	908	74.12	71.56–76.54
	Male friend or partner	13	2.13	0.98–3.28	30	4.86	3.16–6.56	43	3.51	2.58–4.74
	Others	44	7.22	5.16–9.29	57	9.24	6.95–11.53	101	8.24	6.79–9.96
Resident of	The survey city	553	90.80	88.50–93.11	443	71.80	68.24–75.36	996	84.26	82.03–86.27
	Other cities in Jiangsu	25	4.10	2.52–5.68	62	10.05	7.67–12.43	87	7.36	5.97–9.04
	Other Provinces	31	5.09	3.34–6.84	68	11.02	8.54–13.50	99	8.38	6.89–10.14
Condom use with regularpartners in last 3 months	Never	356	61.27	57.30–65.25	276	54.54	50.19–58.90	632	58.14	55.14–61.09
	Sometime	201	34.60	30.72–38.47	180	35.57	31.39–39.76	381	35.05	32.23–37.98
	Always	11	1.89	0.78–3.00	41	8.10	5.72–10.49	52	4.78	3.63–6.27
	Refused	13	2.24	1.03–3.44	9	1.78	0.62–2.93	22	2.02	1.30–3.10
Used condom at lastvaginal sex with regularpartners	Yes	101	17.18	14.12–20.24	113	22.42	18.77–26.08	214	19.60	17.31–22.10
	No	487	82.82	79.76–85.88	391	77.58	73.92–81.23	878	80.40	77.90–82.69
Had sex with FSWsin the past three months	Yes	234	38.81	34.90–42.71	240	38.96	35.10– 42.82	474	38.88	36.15–41.69
	No	369	61.19	57.29–65.10	376	61.04	57.18–64.90	745	61.12	58.31–63.85
Used condom at lastvaginal intercourse with FSWs	Yes	52	21.50	16.28–26.70	60	24.69	19.23–30.15	112	23.09	19.47–27.15
	No	190	78.50	73.30–83.72	183	75.31	69.85–80.77	373	76.91	72.85–80.53
Had sex with casualpartners (excludingFSWs) in last three months	Yes	124	20.80	17.54–24.07	112	18.15	15.10–21.20	236	19.38	17.21–21.73
	No	472	79.20	75.93–82.46	505	81.85	78.80–84.90	982	80.62	78.27–82.79
Used condom at lastvaginal sex withcasual partners(excluding FSWs)	Yes	29	21.01	14.13–24.07	45	36.00	28.50–44.53	74	28.14	22.87–34.05
	No	109	78.99	72.10–85.87	80	64.00	55.47–72.5	189	71.86	65.95–77.13
Ever had sexwith any man	Yes	6	1.03	0.21–1.85	10	1.62	0.62–2.62	16	1.31	0.77–2.16
	No	603	98.97	98.15–99.79	606	98.38	97.38–99.38	1209	98.69	97.84–99.23
Syphilis test result	Positive	110	18.06	15.00–21.13	102	16.53	13.59–19.47	212	17.29	15.24–19.55
	Negative	499	81.94	78.87–85.00	515	83.47	80.53–86.41	1014	82.71	80.45–84.76
HIV test result	Positive	2	0.33	0.00–0.78	4	0.65	0.01–1.28	6	0.49	0.20–1.12]
	Negative	607	99.67	99.22–100.00	614	99.35	98.72–99.99	1220	99.51	98.88–99.80

(N = 1225).

Only about 5% of the study participants reported consistent condom-use with their regular heterosexual partners in the past three months (2% for Yangzhou and 8% for Changzhou)while approximately 82% of the participating MSCs did not use condom during the last vaginal intercourse with their regular partners. (Table 1).

The proportion of study subjects who reported having sex (at least once) with FSWs and non-FSW casual heterosexual partners in the past three months were about 39% and 19% respectively. Meanwhile, for those participants who ever had sex with FSWs, about 77% did not use condom at the last vaginal intercourse with them. This percentage was about 72%for those who ever had sex with other casual partners in the past three months. Besides these, 16 (about 1.31%) of the participants ever had sex with any men. (Table 1).

**Figure 1:**
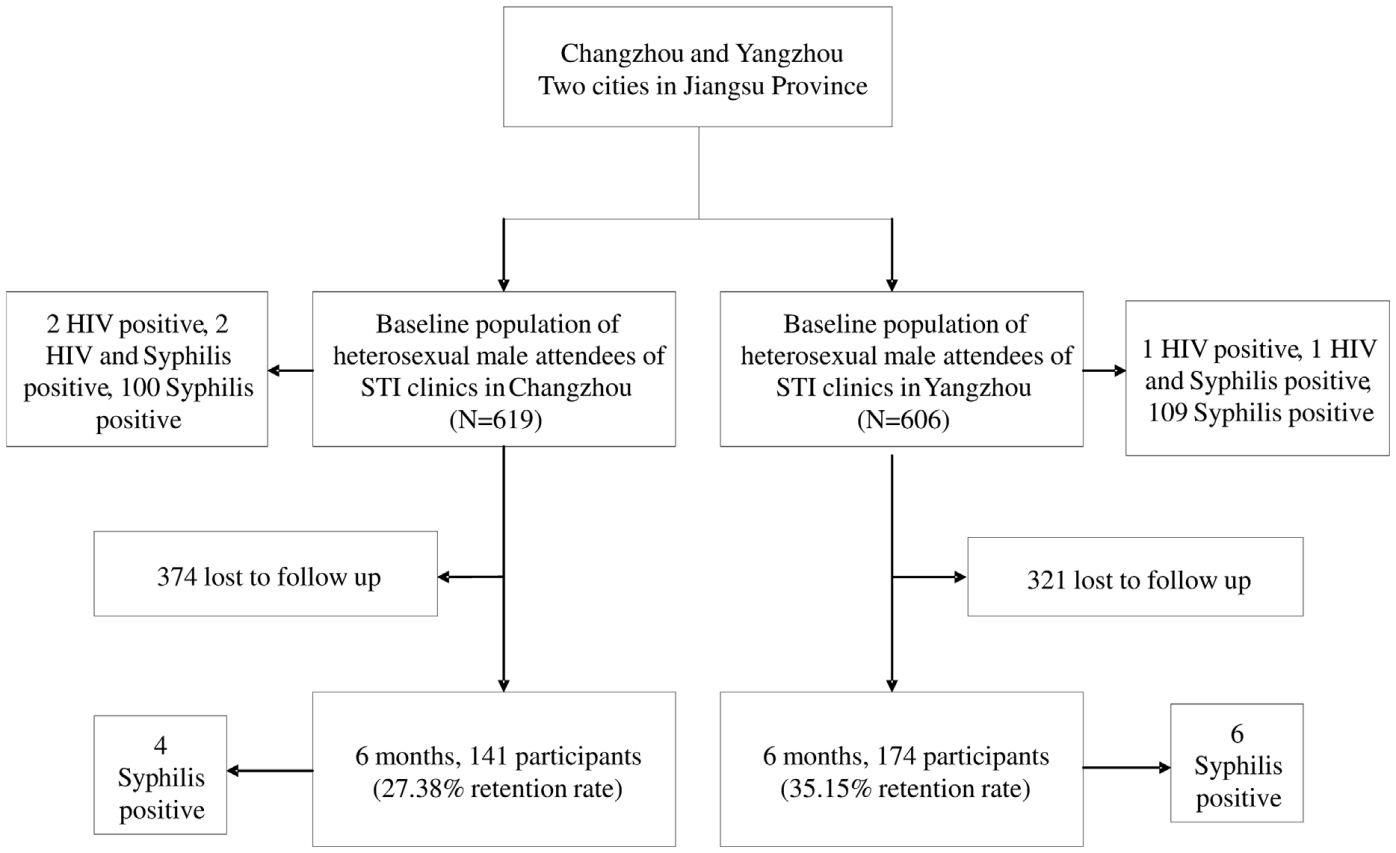
Flow chart of the recruitment of the cohort among heterosexual male attendees of STI clinics (MSC) in Yangzhou and Changzhou cities of Jiangsu Province, China, 2009. (n = 1225).

Figure 1 presents the flow chart of the cohort study in the two target cities. The retention fractions for Yangzhou and Changzhou were 27.38% and 35.15% respectively with 141 and 174 participants attending the 6-month follow up in the respective cities. During the follow up period, no participant was HIV sero-converted, while 10 participants were syphilis sero-converted. The syphilis incidence rates in Yangzhou and Changzhou were 6.53 (95%CI: 0.11–12.95) and 7.76 (95%CI: 1.53–14.00) per 100-person-years respectively, while the overall syphilis incidence rate in these two cities was 7.22 (95%CI: 2.76–11.68) per 100-person-years (Table 2).

**Table 2:** Syphilis incidence rates among heterosexual male attendees of STI clinics (MSC) in Changzhou and Yangzhou cities of Jiangsu Province, China, during 2009–2010.

Disease	City	Follow up (0–6 months)				
		Totalperson-time	Person-time for sero-converted participants	Number of sero-converted participants	Incidence (per 100person-years)	Overall incidence for twocities (per 100 person-years)
Syphilis	Changzhou	62.13	1.76	4	6.53 (0.11, 12.95)	7.22 (2.76, 11,68)
	Yangzhou	79.16	3.67	6	7.76 (1.53, 14.00)	

(N = 1225).

The measured association between syphilis sero-positivity and socio-behavioral factors are presented in Table 3. The bivariate analysis demonstrated that compared to those who where aged between 25 and 40 years, the participants aged 40 or more were at significantly higher risk of syphilis infection (OR = 2.01, 95%CI: 1.47–2.74). Compared to high school educated participants, illiterate and elementary school attendees had higher risk of syphilis infection (OR = 1.52, 95%CI: 1.03–2.23) while those who had college or higher level of education were much less likely to have syphilis (OR = 0.32, 95%CI: 0.17–0.59). Married (OR = 1.53, 95%CI: 1.02–2.32) and divorced/widowed (OR = 2.66, 95%CI: 1.17–6.06)participants had higher likelihood of being syphilis infected, with reference to single subjects. In comparison with the official residents of other cities in Jiangsu Province, dwellers of the sampled cities had significantly higher risk of being syphilis sero-positive (OR = 2.06, 95%CI: 1.14–3.73). The observed differences in the risk of syphilis were not statistically powered enough across the strata of living status to claim any association. While engaging in vaginal intercourse with regular partners in the past three months, compared to the participants who always used condoms, the ones who used never, had higher odds of being syphilis positive (OR = 1.56, 95%CI: 1.01–2.42). Similar results were found for the variables “Not used condom during the last vaginal intercourse with regular partners” (OR = 1.48, 95%CI: 1.04–2.10) and “Had sex with FSWs in the past three months “(OR = 1.42, 95%CI:1.04–1.94). Although condom use while having the most recent vaginal intercourse with FSWs seemed to be protective against syphilis acquisition (OR = 0.52, 95%CI: 0.27–1.02) the analysis did not have sufficient power. HIV sero-positivity status was found to be strongly associated with the likelihood of being positive for syphilis (OR = 4.82, 95%CI: 0.97–24.06).

**Table 3:** Factors correlated with syphilis among heterosexual male attendees of STI clinics (MSC) in Yangzhou and Changzhou cities of Jiangsu Province, China, 2009.

Variables		Crude		Adjusted	
		OR(95%CI)	*P*-value	OR(95%CI)	*P*-value
Age	Less than 25	0.77(0.44–1.36)	0.37	0.79(0.38–1.63)	0.52
	25–40	Ref		Ref	
	40 and above	**2.01(1.47–2.74)**	**<0.001**	**1.64(1.16–2.32)**	**0.01**
Education	Illiteracy or elementary school	**1.52(1.03–2.23)**	**0.03**	1.36(0.90–2.07)	0.14
	Junior or senior high school	Ref		Ref	
	College or above	**0.32(0.17–0.59)**	**<0.001**	**0.36(0.19–0.67)**	**<0.001**
Marital Status	Single	Ref		Ref	
	Married	**1.53(1.02–2.32)**	**0.04**	0.60(0.28–1.28)	0.19
	Divorced or widowed	**2.66 (1.17–6.06)**	**0.02**	1.29(0.51–3.28)	0.59
Living status	Living alone	Ref		Ref	
	With heterosexual partners	1.31(0.84–2.05)	0.24	1.36(0.66–2.79)	0.40
	With homosexual partners	0.43(0.12–1.48)	0.18	0.51(0.14–1.82)	0.30
	Others	0.84(0.41–1.72)	0.64	0.92(0.42–1.98)	0.82
Resident	The survey city	**2.06(1.14–3.73)**	**0.02**	1.79(0.96–3.35)	0.07
	Other cities in Jiangsu Province	Ref		Ref	
	Other Provinces	1.62(0.73–3.58)	0.23	1.63(0.72–3.70)	0.24
Condom use with regular partnersin past 3 months	Never	**1.56(1.01–2.42)**	**0.05**	1.09(0.57–2.08)	0.79
	Sometime	1.14(0.70–1.84)	0.60	1.03(0.57–1.87)	0.93
	Always	Ref		Ref	
Used condom during last vaginal sexwith regular partners	No	**1.48(1.04–2.10)**	**0.03**	1.14(0.69–1.87)	0.61
	Yes	Ref		Ref	
Female partners had symptoms ofSTIs in the past year	Yes	0.85(0.63–1.14)	0.28	0.76(0.55–1.04)	0.08
	No	Ref		Ref	
Had sex with FSWs in the past three months	Yes	**1.42(1.04–1.94)**	**0.03**	**1.42(1.01–2.00)**	**0.04**
	No	Ref		Ref	
Used condom at last vaginal intercoursewith FSWs	Yes	Ref		Ref	
	No	1.92(0.98–3.70)	0.06	1.19(0.57–2.50)	
Had sex with casual partners (not include FSWs)in the past three months	Yes	0.87(0.60–1.27)	0.47		
	No	Ref			
Ever had sex with any man	Yes	0.76(0.39–1.46)	0.40		
	No	Ref			
HIV test result	Positive	4.82(0.97–24.06)	0.06	**6.29(1.07–37.07)**	**0.04**
	Negative	Ref		Ref	

Bold numerical values indicate results for which *P*-value was less than 0.05. (n = 1225).

In the multivariate analysis, after adjustment for other variables, age-group of 40 years or more(OR = 1.64, 95%CI: 1.16–2.32), having sex with FSWs in the past three months (OR = 1.42, 95%CI: 1.01–2.00) and being positive for HIV infection OR = 6.29(95%CI: 1.07–37.07)were still associated with higher odds of syphilis infection compared to the respective reference groups. Alike the univariate analysis, official residents of the sampled cities seemed to have higher propensity of having syphilis infection, even though the result marginally lacked power (OR = 1.79, 95%CI: 0.96–3.35). Meanwhile, similar to the unadjusted analysis, the participants who had college level or higher education did exhibit significantly less chance of syphilis infection (OR = 0.36, 95%CI: 0.19–0.67) after adjustment for other correlates.

## Discussion

This cohort study conducted in two cities in Jiangsu Province, China provided information on the prevalence of HIV and syphilis, incidence of syphilis and developed insight regarding the demographic and behavioral correlates of syphilis infection among heterosexual male clients of STI-clinics.

The observed prevalence of HIV among the participating MSCs was 0.49%, lower than the prior findings among male clients of female sex workers in the border area of the Yunnan province of southern China [12], in Sichuan province [19] and in Tijuana, Mexico [14]. However, this prevalence was higher than the findings among male STI-clinic clients in USA [20]. Meanwhile, the overall syphilis prevalence for the participants who attended the baseline survey was 17.29% (18.06% for Yangzhou and 16.53% for Changzhou). These syphilis prevalence rates were much higher than the observed prevalence among male clients of female sex workers in prior studies conducted in border region of Yunnan [12] and Sichuan, China [19].

The overall syphilis incidence rate in our study was 7.22 per 100-person-years, which may also be considered as alarmingly high.

Increasing burden of syphilis is currently a major public health concern in China as the prevalence in general population has peaked to 20.0 cases per 100,000 in 2010 [5]. Keeping in mind this recent resurgence [21], the observed high prevalence and incidence among MSCs probably indicated towards the expanding epidemic of syphilis in this country. Higher incidence and prevalence of this disease in this bridge population may further complicate HIV/other STI epidemic situations, since presence of syphilis may facilitate transmission of these diseases [22]. Thus targeted interventions to control the spread of syphilis within and from MSC population seem to be the need of the hour.

The retention fractions for Yangzhou and Changzhou were about 27% and 35% respectively. There were several potential reasons for such a lower retention pattern.(1) Yangzhou and Changzhou are two cities famous for activities of entertainment including commercial sex. Many male residents from the surrounding areas and other parts of the country visit these cities for fun and many of them engage in commercial sex, which might have enhanced the mobility of the clients and in turn diminished the retention. (2) More than three quarters of the participants were married, majority of whom were likely to be afraid of the possibility that their identity as STI patients might be divulged to their regular sexual partner. Hence despite of sincere efforts to motivate all the participants at baseline to participate in the follow up, a considerable proportion of those married clients were likely to be lost to follow up. (3) Potential reasons of low retention could also be that STI-clinic attendees, irrespective of their STI test results at baseline, if cured of their STIs or related symptoms, were unlikely to return for further follow up and testing [23]. Combining all of these together, the lower retention fraction in this population was not surprising.

Among the study participants, approximately 77% were married, 74% were living with their heterosexual partners and about 60% never used condom while engaging in vaginal intercourse with their regular partners. These patterns combined with the higher syphilis prevalence and incidence rates among the study subjects together indicated that MSCs in these two cities are likely to play a potentially dangerous bridging role in the transmission of HIV and other STIs to their female partners [24]. Thus targeted intervention programs may be urgently required to prevent syphilis transmission from MSCs to general population especially their heterosexual partners in this part of China.

In our study, only about 15% participants attended college or the other higher education. Lower education attainment may well be a potential correlate of the risk of syphilis [25]. The results of our univariate and multivariate analysis also demonstrated that higher education was associated with lower likelihood of being sero-positive for syphilis. This corroborated with the findings of some previous studies that lower education was positively associated with increased proportion of high risk sexual behaviors [26], [27], which in turn might have lead to higher chance of syphilis acquisition.

It was observed that about 84% subject were the official residents of the sampled city (Yangzhou and Changzhou) and participating MSCs from these two metro cities seemed to be at higher risks of syphilis infection, compared to the those from other cities in Jiangsu Province. These results were different from prior research among MSM, which demonstrated that non-resident MSM in Chinese cities were more likely to engage in high-risk sexual behaviors and acquire HIV/STIs infection than residents MSM [28], [29]. The logical mechanisms behind these dynamics are needed to be further explored in order to establish effective intervention strategies targeting the MSCs in China.

Both univariate and multivariate analysis indicated that those who had sex with FSWs in the past three months had higher risk of being infected with syphilis. Similar findings were reported from another study in China, in which the clients of the FSWs were more likely to report STIs [30]. In contradiction with previous observations among MSM, participating MSCs who reported to have sex with men or recent sex with non-FSW casual partners did not exhibit any statistically significant higher likelihood of syphilis acquisition [13]. Detailed explorations are likely to be required to develop insight into these counter-intuitive findings involving this understudied population of MSC.

The results of our study also demonstrated that the participants who did use condom during their last vaginal intercourse with FSWs were less likely to be syphilis positive. While condom use was very likely to have a true association with protection against syphilis, reverse causation could also be one potential explanation. Those having symptomatic sexually transmitted infections (including syphilis) were more likely to use and be asked to use condoms while engaging in vaginal sex with FSWs.

Similar to several previous studies in other high-risk populations, being HIV positive was found to be strongly associated with syphilis sero-positivity [31]. However, we must admit that it was difficult to draw an inference on the direction of this association based on our cross-sectional baseline data.

To the best of our knowledge, this was the first cohort study among MSCs in China and the initial effort to measure the incidence of STIs in this population. Besides this, the other strengths of our study included large sample size and having more than one sites of observation. To avoid interviewers’ bias, a uniform protocol was followed at each site and all the interviewers were trained together.

Like other observational studies, our results also had some limitations. Due to its self-reported and face-to-face interview nature, most of our data were vulnerable to suffer from social desirability bias that, in turn, might have lead to exposure or confounder misclassifications. As HIV and syphilis test results were conveyed after the interview, these misclassifications were likely to be non-differential. However, possibility of differential misclassification could not be ruled out completely because some participants might have been symptomatic or aware of their disease status before participation. Also, despite of excluding the subjects who reported to have sex with men in last 3 months, there were possibilities that our study population of MSCs might have included some STI affected MSMs due to non-reporting of their MSM behavior while attending the clinic and this might have resulted in overestimation. But these misclassifications, if present were likely to be of lower magnitude because these subjects attended the clinics to seek healthcare services; hence they were likely to be truthful.

Selection bias might also be considered as a threat to the validity of the cohort part of our study as a large proportion of the participants at baseline were lost to follow up during the course of the study. This higher loss to follow up might have resulted in potential underestimation of the incidence rates measured in our study, as those who did not abide by the study protocol were likely to have higher risk behaviors than their adherent counterparts. This was potentially proven by our study, which revealed that the participants who were lost to follow up hardly used condoms while engaging in commercial sex with FSWs in the past three months (data not shown here). Short follow up period and chances of residual confounding due to unknown and unmeasured confounders were the other potential limitations of our study. Additionally, patients with positive syphilis or HIV test at baseline were not followed up. Hence any new occurrence of HIV among those who had syphilis at baseline and new syphilis infections among the HIV positives at baseline were not included in our estimates. This might have generated some potential for underestimation. Also, we could not compare the baseline characteristics of the participants who were lost-to-follow-up with those who were not. Thus evaluation of the potential influences of the large amount of lost-to-follow-up on the incidence rates was not possible. There was also a possibility that some subjects with high-risk exposure might have attended the clinic for testing immediately. Thus participants sero-converted during a particular follow up period might have been in the window period during the corresponding baseline testing prior to the follow-up period, resulting in underestimation of the existing cases at baseline and overestimation of the incident cases thereafter. But for HIV, we consider this possibility to be very little, as most of these cases would have been identified via RNA testing.

The prevalence and incidence of syphilis was observed to be high among MSCs in Jiangsu. Syphilis infected persons in this group may serve as a bridge population in transmitting this STI to their spouses, FSWs, other regular and casual sexual partners while engaging in improperly protected vaginal sex. Our results strongly suggest that effective intervention strategies targeting the correlates of syphilis infection are urgently called for in this province.

## References

[1] Wu Z, Wang Y (2010) Introduction: China meets new AIDS challenges. JAIDS Journal of Acquired Immune Deficiency Syndromes 53: S1–S3. 20104098 10.1097/QAI.0b013e3181c7d379

[2] Tucker JD, Chen X-S, Peeling RW (2010) Syphilis and social upheaval in China. New England Journal of Medicine 362: 1658–1661. 20445179 10.1056/NEJMp0911149

[3] Chen ZQ, Zhang GC, Gong XD, Lin C, Gao X, et al (2007) Syphilis in China: results of a national surveillance programme. Lancet 369: 132–138. 17223476 10.1016/S0140-6736(07)60074-9 PMC7138057

[4] Cohen MS, Henderson GE, Aiello P, Zheng H (1996) Successful eradication of sexually transmitted diseases in the People’s Republic of China: implications for the 21st century. J Infect Dis 174 Suppl 2S223–229. 8843252 10.1093/infdis/174.supplement_2.s223

[5] Tucker JD, Cohen MS (2011) China’s syphilis epidemic: epidemiology, proximate determinants of spread, and control responses. Current opinion in infectious diseases 24: 50. 21150594 10.1097/QCO.0b013e32834204bf PMC3103765

[6] Chen Z-Q, Zhang G-C, Gong X-D, Lin C, Gao X, et al (2007) Syphilis in China: results of a national surveillance programme. The Lancet 369: 132–138. 10.1016/S0140-6736(07)60074-9 PMC7138057 17223476

[7] Benzaken AS, García EG, Sardinha JCG, Junior JCD, Peeling R (2007) Rapid tests for diagnosing syphilis: validation in an STD clinic in the Amazon Region, Brazil Testes rápidos para diagnóstico de sífilis: validação em clínica de DST na Região Amazônica, Brasil. Cad saúde pública 23: S456–S464. 17992351 10.1590/s0102-311x2007001500013

[8] Baffi CW, Aban I, Willig JH, Agrawal M, Mugavero MJ, et al (2010) New syphilis cases and concurrent STI screening in a southeastern US HIV clinic: a call to action. AIDS patient care and STDs 24: 23–29. 20095902 10.1089/apc.2009.0255 PMC2859761

[9] Wong SP, Yin Y-P, Gao X, Wei W-H, Shi M-Q, et al (2007) Risk of syphilis in STI clinic patients: a cross-sectional study of 11 500 cases in Guangxi, China. Sexually Transmitted Infections 83: 351–356. 17591664 10.1136/sti.2007.025015 PMC2659023

[10] Abdullah AS, Fielding R, Hedley AJ, Luk YK (2002) Risk factors for sexually transmitted diseases and casual sex among Chinese patients attending sexually transmitted disease clinics in Hong Kong. Sexually transmitted diseases 29: 360–365. 12035027 10.1097/00007435-200206000-00009

[11] Van Dam JC, Holmes KK (2000) STD prevention: effectively reaching the core and a bridge population with a four-component intervention. Sexually transmitted diseases 27: 9–11. 10654861 10.1097/00007435-200001000-00002

[12] Reilly KH, Wang J, Zhu Z, Li S, Yang T, et al (2012) HIV and Associated Risk Factors Among Male Clients of Female Sex Workers in a Chinese Border Region. Sexually transmitted diseases 39: 750–755. 23007705 10.1097/OLQ.0b013e31825f7af7 PMC3458308

[13] Ribeiro D, Rezende EF, Pinto VM, Pereira GFM, Miranda AE (2012) Prevalence of and risk factors for syphilis in Brazilian armed forces conscripts. Sexually Transmitted Infections 88: 32–34. 22037855 10.1136/sextrans-2011-050066

[14] Goldenberg SM, Strathdee SA, Gallardo M, Nguyen L, Lozada R, et al (2011) How important are venue-based HIV risks among male clients of female sex workers? A mixed methods analysis of the risk environment in nightlife venues in Tijuana, Mexico. Health & place 17: 748–756. 21396875 10.1016/j.healthplace.2011.01.012 PMC3092829

[15] Palacios R, Jiménez-Onate F, Aguilar M, Galindo MJ, Rivas P, et al (2007) Impact of syphilis infection on HIV viral load and CD4 cell counts in HIV-infected patients. JAIDS Journal of Acquired Immune Deficiency Syndromes 44: 356–359. 17159654 10.1097/QAI.0b013e31802ea4c6

[16] Li D, Wang L, Lin W, Li P, Guo W, et al (2014) HIV and syphilis infections among street-based female sex workers in China, 2010–2012. Chin Med J (Engl) 127: 707–711. 24534227

[17] Lauritsen J, Bruus M (2003) EpiData (version 3). A comprehensive tool for validated entry and documentation of data Odense: EpiData Association 2004.

[18] Institute S (1996) The SAS system for Windows. SAS Institute Cary, NC.

[19] Yang C, Latkin C, Luan R, Wang C, Nelson K (2010) HIV, syphilis, hepatitis C and risk behaviours among commercial sex male clients in Sichuan province, China. Sexually Transmitted Infections 86: 559–564. 20826867 10.1136/sti.2009.041731 PMC4487621

[20] Xu F, Stoner BP, Taylor SN, Mena L, Martin DH, et al (2013) “Testing-Only” Visits: An Assessment of Missed Diagnoses in Clients Attending Sexually Transmitted Disease Clinics. Sexually transmitted diseases 40: 64–69. 23254118 10.1097/OLQ.0b013e31826f32f3

[21] Tucker JD, Yin YP, Wang B, Chen XS, Cohen MS (2011) An expanding syphilis epidemic in China: epidemiology, behavioural risk and control strategies with a focus on low-tier female sex workers and men who have sex with men. Sex Transm Infect 87 Suppl 2ii16–18. 22110145 10.1136/sti.2010.048314 PMC3306605

[22] Freeman EE, Orroth KK, White RG, Glynn JR, Bakker R, et al (2007) Proportion of new HIV infections attributable to herpes simplex 2 increases over time: simulations of the changing role of sexually transmitted infections in sub-Saharan African HIV epidemics. Sexually Transmitted Infections 83: i17–i24. 17405782 10.1136/sti.2006.023549

[23] Guan J, Wu Z, Li L, Lin C, Rotheram-Borus MJ, et al (2009) Self-reported sexually transmitted disease symptoms and treatment-seeking behaviors in China. AIDS patient care and STDs 23: 443–448. 19519228 10.1089/apc.2008.0204 PMC2856433

[24] Qian Z, Vermund S, Wang N (2005) Risk of HIV/AIDS in China: subpopulations of special importance. Sexually Transmitted Infections 81: 442–447. 16326842 10.1136/sti.2004.014258 PMC1745065

[25] Hampton MC, Halkitis PN, Storholm ED, Kupprat SA, Siconolfi DE, et al.. (2012) Sexual risk taking in relation to sexual identification, age, and education in a diverse sample of African American men who have sex with men (MSM) in New York City. AIDS and Behavior: 1–8. 10.1007/s10461-012-0139-8 PMC12335616 22298339

[26] Safika I, Johnson TP, Levy JA (2011) A venue analysis of predictors of alcohol use prior to sexual intercourse among female sex workers in Senggigi, Indonesia. International Journal of Drug Policy 22: 49–55. 20956075 10.1016/j.drugpo.2010.09.003 PMC3031741

[27] Strathdee SA, Hogg RS, Martindale SL, Cornelisse PG, Craib KJ, et al (1998) Determinants of sexual risk-taking among young HIV-negative gay and bisexual men. JAIDS Journal of Acquired Immune Deficiency Syndromes 19: 61–66. 10.1097/00042560-199809010-00010 9732071

[28] Zou H, Wu Z, Yu J, Li M, Ablimit M, et al (2010) Sexual risk behaviors and HIV infection among men who have sex with men who use the internet in Beijing and Urumqi, China. Journal of acquired immune deficiency syndromes (1999) 53: S81. 20104115 10.1097/QAI.0b013e3181c7dd2b PMC2818820

[29] Tang W, Huan X, Mahapatra T, Tang S, Li J, et al.. (2013) Factors Associated with Unprotected Anal Intercourse Among Men Who Have Sex with Men: Results from a Respondent Driven Sampling Survey in Nanjing, China, 2008. AIDS and Behavior: 1–8. 10.1007/s10461-013-0413-4 23334360

[30] Huang Z, Wang W, Martin M, Nehl E, Smith B, et al (2011) “Bridge population”: sex workers or their clients?–STI prevalence and risk behaviors of clients of female sex workers in China. AIDS care 23: 45–53. 21660750 10.1080/09540121.2010.507759 PMC8103540

[31] Xiao Y, Sun J, Li C, Lu F, Allen KL, et al (2010) Prevalence and correlates of HIV and syphilis infections among men who have sex with men in seven provinces in China with historically low HIV prevalence. JAIDS Journal of Acquired Immune Deficiency Syndromes 53: S66–S73. 20104113 10.1097/QAI.0b013e3181c7db43

